# Person- and Task-Centered Mealtime Care: Impact on Positive, Neutral, and Challenging Behaviors in People With Dementia

**DOI:** 10.1093/geroni/igab046.1559

**Published:** 2021-12-17

**Authors:** Wen Liu, Kristine Williams, Melissa Batchelor, Yelena Perkhounkova, Maria Hein

**Affiliations:** 1 The University of Iowa College of Nursing, Iowa City, Iowa, United States; 2 University of Kansas School of Nursing, Kansas City, Kansas, United States; 3 George Washington University, Washington, D.C., District of Columbia, United States; 4 University of Iowa, Iowa City, Iowa, United States

## Abstract

Mealtime is an important daily activity to ensure intake. Person-centered and task-centered care may influence individual positive, neutral, and challenging mealtime behaviors. Yet, little work has fully examined their relationships. This study aimed to examine the association between person-centered and task-centered care approaches and individuals’ positive, neutral, and challenging mealtime behaviors. This secondary analysis of 110 videotaped mealtime observations involved 29 staff and 25 residents with dementia (42 unique staff-resident dyads) in 9 nursing homes. Videos were coded using the refined Cue Utilization and Engagement in Dementia mealtime video-coding scheme. Logistic regression models were fit to four dependent variables representing resident mealtime behaviors: 1) positive/neutral behaviors (nonverbal), 2) positive utterances (verbal), 3) functional impairments (nonverbal), and 4) resistive behaviors (verbal and nonverbal). Independent variables were staff person-centered care modifications (nonverbal), person-centered utterances (verbal), and task-centered behaviors (verbal and nonverbal). Covariates included resident age, gender, eating function, and video duration. Resident positive utterances were associated with staff person-centered care utterances (OR =1.38, 95% CI = 1.09,1.76). Resident functional impairments were associated with staff person-centered care modifications (OR=1.33, 95% CI=1.02, 1.74) and fewer staff person-centered utterances (OR=0.81, 95% CI=0.66, 1.00). Resident resistive behaviors were associated with more staff person-centered utterances (OR=1.65, 95% CI=1.18, 2.31). Findings provided preliminary information supporting the role of staff person-centered care on resident positive and challenging mealtime behaviors. Findings inform use of verbal and nonverbal person-centered care strategies to improve positive communication and reduce challenging behaviors during mealtime in people with dementia.

